# “Zebrafishing” for Novel Genes Relevant to the Glomerular Filtration Barrier

**DOI:** 10.1155/2013/658270

**Published:** 2013-09-11

**Authors:** Nils Hanke, Lynne Staggs, Patricia Schroder, Jennifer Litteral, Susanne Fleig, Jessica Kaufeld, Cornelius Pauli, Hermann Haller, Mario Schiffer

**Affiliations:** ^1^Division of Nephrology, Hannover Medical School, Carl-Neuberg-Strße 1, 30625 Hannover, Germany; ^2^Mount Desert Island Biological Laboratory, P.O. Box 35, Old Bar Harbor Road, Salisbury Cove, ME 04672, USA

## Abstract

Data for genes relevant to glomerular filtration barrier function or proteinuria is continually increasing in an era of microarrays, genome-wide association studies, and quantitative trait locus analysis. Researchers are limited by published literature searches to select the most relevant genes to investigate. High-throughput cell cultures and other *in vitro* systems ultimately need to demonstrate proof in an *in vivo* model. Generating mammalian models for the genes of interest is costly and time intensive, and yields only a small number of test subjects. These models also have many pitfalls such as possible embryonic mortality and failure to generate phenotypes or generate nonkidney specific phenotypes. Here we describe an *in vivo* zebrafish model as a simple vertebrate screening system to identify genes relevant to glomerular filtration barrier function. Using our technology, we are able to screen entirely novel genes in 4–6 weeks in hundreds of live test subjects at a fraction of the cost of a mammalian model. Our system produces consistent and reliable evidence for gene relevance in glomerular kidney disease; the results then provide merit for further analysis in mammalian models.

## 1. Introduction

Chronic kidney disease (CKD) is a national and world healthcare priority. CKD is rarely detected early enough in patients, typically leads to kidney failure, and frequently requires therapy through dialysis or transplantation. Proteinuria is one of the clinical hallmarks when diagnosing CKD. The need for therapies is considerable; presently, we are only able to treat the sequel of CKD, hypertension, and metabolic disease. In order to discover new therapeutic strategies, we have to understand the molecular mechanisms of the disease and identify novel targets. 

The generation of a murine model used to identify mechanisms and novel genes relevant to CKD and proteinuria is time consuming, is very costly, yields only a small number of test subjects, and can have other serious shortcomings such as embryonic mortality and failure to generate phenotypes, or generate nonkidney specific phenotypes. However, zebrafish are an ideal model to screen novel genes relevant to glomerular filtration barrier function or proteinuria. Using zebrafish, we are able to screen entirely novel genes in 4–6 weeks in hundreds of live test subjects at a fraction of the cost of a mammalian model. Zebrafish develop from a fertilized egg to free-swimming larvae in only 48 hours and develop a fully functional kidney unit within 72 hours, and effects can be monitored within 2-3 days after fertilization. Protein production in zebrafish larvae can easily be influenced by specific gene knockdown or overexpression techniques. However, proof of specific and definite alteration of protein expression level has to be given for each experiment. This paper covers these basic techniques and describes the zebrafish model as a simple and fast screening system to identify genes relevant for the integrity of the glomerular filtration barrier. Using our approach the question of relevance for a particular gene can be answered within a time frame of 4–6 weeks, and this evidence can be the basis for further analysis in rodent models or human tissues.

## 2. Morpholino or gripNA Technology

### 2.1. Using Morpholino Techniques to Knockdown Genes

Morpholino antisense oligos, developed by Dr. James Summerton, are synthetic short nucleic acid analogs. They are used to knockdown genes by blocking translation of gene-specific mRNA [1]. A morpholino consists of 25 subunits; each subunit consists of a nucleic acid base linked to a morpholine ring via a nonionic phosphorodiamidate, also known as *phosphorodiamidate morpholino oligo* (PMO). The antisense oligo is rendered water soluble and immune to enzymatic degradation due to the backbone structure of morpholine moiety linked to a phosphorodiamidate [2]. Morpholino antisense oligos have strong RNA binding affinity, and low production costs offer the following additional advantages over other antisense oligonucleotides: biological stability, high specificity, and high antisense efficacy.

 As the morpholino concentration dilutes with every cell division the morpholino remains stable, and the effect of morpholino gene knockdown becomes transient.

Yan et al. [3] used a *splice-donor morpholino* against *sox9a* and found an 80% inhibition rate 28 hours postfertilization (hpf); this decreased to 45% at 96 hpf. Morpholinos are the most widely used tool today in zebrafish loss-of-function studies.

Alternatively gripNA, a technique based on peptide nucleic acids (PNAs), can be used as antisense strategy. PNAs are DNA analogs where the four nucleosides, adenine (A), thymine (T), guanine (G), and cytosine (C), are attached to an N-(2-aminoethyl)-glycine backbone rather than to a deoxyribose phosphate backbone. Use of PNAs was limited by lack of affinity, a problem which has been recently overcome by introduction of negative charges to enhance binding [4–8].

### 2.2. ATG-Blocker versus Splice Donor

The primary function of a morpholino is to block translation or to act as a splicedonor. To prevent translation, the translational blocking morpholinos bind close to the translational start site of the complementary mRNA in the 5′-untranslated region (UTR) [9]. *Splice-donor morpholinos* alter premRNA splicing by targeting binding sites for small nuclear ribonucleoproteins (snRNPs) or splicejunctions [10, 11]. An mRNA that lacks an exon or has an inserted intron is produced when a morpholino binds to either site, and the spliceosome fails to recognize the splice junction sequence or snRNP-binding site. 

A frameshift in the mRNA results when sections of nucleotides are removed or inserted; a shift in the reading frame knocks down gene expression. Frameshifts change the amino acid added by the ribosome that either no longer codes for the original protein or introduces a premature termination codon that triggers degradation of the mRNA.

### 2.3. Morpholino Injection in Embryos

Morpholino microinjection experiments to knockdown genes in zebrafish embryos were first used in 2000 [12, 13]. Embryos are obtained by natural spawning of breeding pairs of adult zebrafish. The required equipment needed for microinjection are a microinjector (e.g., Nanoject, Drummond Scientific, PA), a three-dimensional micromanipulator, and a stereo microscope. Microinjection needles are prepared by pulling fine glass capillaries with a micropipette puller; the resulting tip should be about 0.05 mm [14]. Diluted morpholino stock solution (50–250 *μ*M) in morpholino dilution buffer (e.g., 200 mM KCl, 5 mM HEPES, pH 7,0, and 5 *μ*g/mL phenol red) [14] is injected using a glass microinjection needle. To establish an experimental control, usually a scrambled oligonucleotide morpholino is injected into a separate group of embryos. Since zebrafish produce eggs of varying quality an uninjected group of embryos are set aside from each clutch to monitor their health and development. 

Stuart et al. described the first successful zebrafish embryo microinjection in 1988 [15]. One to two cell stage fish embryos still in their chorions are placed in a 1.5% agarose injection mold and are aligned by rotating the germinal disc roughly parallel to the trough such that the developing embryo is clearly visible. The tip of the micromanipulator needle is placed into the yolk sack directly beneath the embryo and penetrates the chorion at a 45° angle. Approximately 2.5–5 nL of morpholino buffer solution is injected into each egg. After injection the embryos are transferred into a clean dish with an embryo raising medium (ERM) [14]. Embryos develop in an incubator at 28.5°C and are checked for viability and cleaned frequently.

### 2.4. Morpholino Injection in Adult Fish

In theory, morpholinos injected into any cell of the developing zebrafish embryo should be as effective as the one-cell stage; however, due to limitations with the morpholinos ability to penetrate the cell membrane this technique is less successful in adult fish.

Morpholino injection methods were first described by transfecting DNA [16–20] and then used in chick embryo with square pulse electroporation [21–23]. This method was adopted by regeneration research for adult axolotl [24] and adult zebrafish in fin regeneration studies [25, 26]. In the fin regeneration studies, a 3′-fluorescein-labeled morpholino is microinjected into the regeneration zone of the adult zebrafish fin directly following amputation and is then treated with square wave electroporation. Fluorescence microscopy detects the morpholino in the tissue and demonstrates that electroporated morpholino was transferred into the cells and regeneration was impaired. However, the uptake of morpholinos into adult tissues worked only in damaged muscle or directly around the site of injection; transport across the cell membrane in systemic delivery of nonmodified morpholinos was poor.

Recently, *vivo-morpholinos* were developed, to make morpholinos a more useful tool in adult tissues. *Vivo-morpholinos* coupled to an artificial molecular transport system allow for easier cellular morpholino uptake [27]. The delivery system consists of eight guanidine head groups on two of three side chains of triazine, and the morpholino is bound to the third side chain [28].

Transgenic mice ubiquitously express EGFP pre-mRNA that contains an aberrantly spliced intron. An intravenous injection of a *vivo-morpholino* that corrects for this splicevariant in mice led to a >90% correction in the mRNAsplice in liver, colon, small intestine, muscle, diaphragm, and kidney, whereas an intravenous injection of a nonmodified morpholino had only a <10% correction (both injected at a dose of 12.5 mg/kg/day for 4 consecutive days) [28]. In tissues from brain, heart, lung, and spleen the splice correction was lower than 30% after *vivo-morpholino* intravenous injection, and intraperitoneal injection led to an even lower splice correction level than intravenous injection. *Vivo-morpholinos* are a potent tool for gene knockdown in adult tissues using multiple applications that are illustrated in the following examples: an intravenous injection of a *vivo-morpholino* targeting the coagulation factor, Von-Willebrand-Factor, led to a bleeding phenotype [29], and a *sox11b* targeted *vivo-morpholino*, administered locally that delayed regeneration and repair in a zebrafish model with spinal cord injury [30]. Thus, *vivo-morpholinos* seem to be a stable solution for genetic manipulation in adult tissues. 

## 3. Injection of mRNA

A widely used morpholino specificity control or to rescue a morpholino-induced phenotype, mRNA is microinjected encodes the morpholino-targeted gene (e.g., in [31–33]). The mRNA is designed so that the morpholino binding site is absent and is coinjected with the morpholino or injected separately in a second injection. To produce an mRNA control, the gene of interest is cloned into a vector that transcribes mRNA from one promoter and also transcribes from another promoter in the opposite direction (antisense) as control. Alternatively, a flipped transcript is cloned into a second vector.

This strategy works with zebrafish mRNA as well as with murine or human mRNA if the gene is highly conserved (cross-species rescue) and enables the user to test if mutant forms of the transcript are rescued to a similar extent. Thus, this system is a great tool to analyze also if a mutation in the gene of interest has biological relevance. 

Injecting mRNA into zebrafish embryos has also been used for nearly two decades to understand effects of gene overexpression (e.g., in [34, 35]). The mRNA coding for a gene of interest is produced synthetically (*in vitro* translation) and is injected at varying concentrations into zebrafish eggs at the 1-2 cell stage; then the translation of the mRNA produces higher amounts of the encoded protein. This technique can also be used to test specific mutations in a protein, and compared to wildtype mRNA this can be used to prove *in vivo* relevance for a point mutation.

## 4. Injection of MicroRNAs

MicroRNAs are highly conserved throughout vertebrates; thus, specific effects of microRNAs identified in mice or human tissues can be tested immediately in the zebrafish system [36]. MicroRNA injection can also be used in rescue experiments, for example, in a study on the role of *miR-430* on zebrafish brain morphogenesis. Injection of *miR-430* in an MZ*dicer*-mutant zebrafish failed to produce endogenous *miR-430* and rescued the mutant phenotype [37]. Injecting siRNA and dsRNA to knockdown genes in zebrafish results in multiple nonspecific defects and is not a preferred method in zebrafish research (details reviewed in [38]). However, they can be used in other animal models to verify a well-defined phenotype [36]. 

For the aforementioned techniques, it is important to verify that the gene expression was altered in the intended manner. Regardless of technique—mRNA, morpholino, or peptide nucleic acid injection—the primary purpose is to demonstrate the appropriate change in protein level. If an antibody is available for the protein of interest, for determination of morpholino efficacy either a western blot from zebrafish protein fry extracts or whole mount immunohistochemistry staining can be performed [39, 40]. If an antibody is unavailable, a transgenic or coinjected mRNA with 5′UTR of the gene of interest upstream of a marker or epitope tag (e.g., hemagglutinin or GFP) could be used to assess the level of knockdown.

Alternatively, RT-PCR can be used to demonstrate morpholino efficacy through either a decrease in the level of mRNA from initiation of nonsense mediated decay or a change in size of the transcript from inclusion of an intron or exclusion of an exon. 

## 5. Analysis of Renal Phenotypes: The Specificity of Generalized Edema

A first hint of a renal phenotype is the development of generalized edema of the zebrafish larvae. However, edema is sometimes considered to be nonspecific, since a small percentage of wildtype fish develops mild edema as well as a sign of developmental defects. In the following we describe how edema in a fish embryo can be interpreted as part of a specific renal phenotype and which pitfalls have to be considered. After injecting a morpholino or mRNA into the 1-2 cell stage zebrafish embryos (as described above), larval development is monitored for 120 hours postfertilization (hpf), and phenotype development and mortality are recorded. For uninjected wildtype and control (scrambled oligonucleotide) injected embryos, the percentage of fish that develop severe generalized edema should be significantly lower. If a high percentage (>40%) of embryos develop edema in the knockdown or overexpression group within the first 120 hpf, this could be a first indication of a renal phenotype. In contrast, nonspecific edema can be observed in small percentages (<3–5%) of uninjected genetically unmodified embryos. 

**Figure 1:**
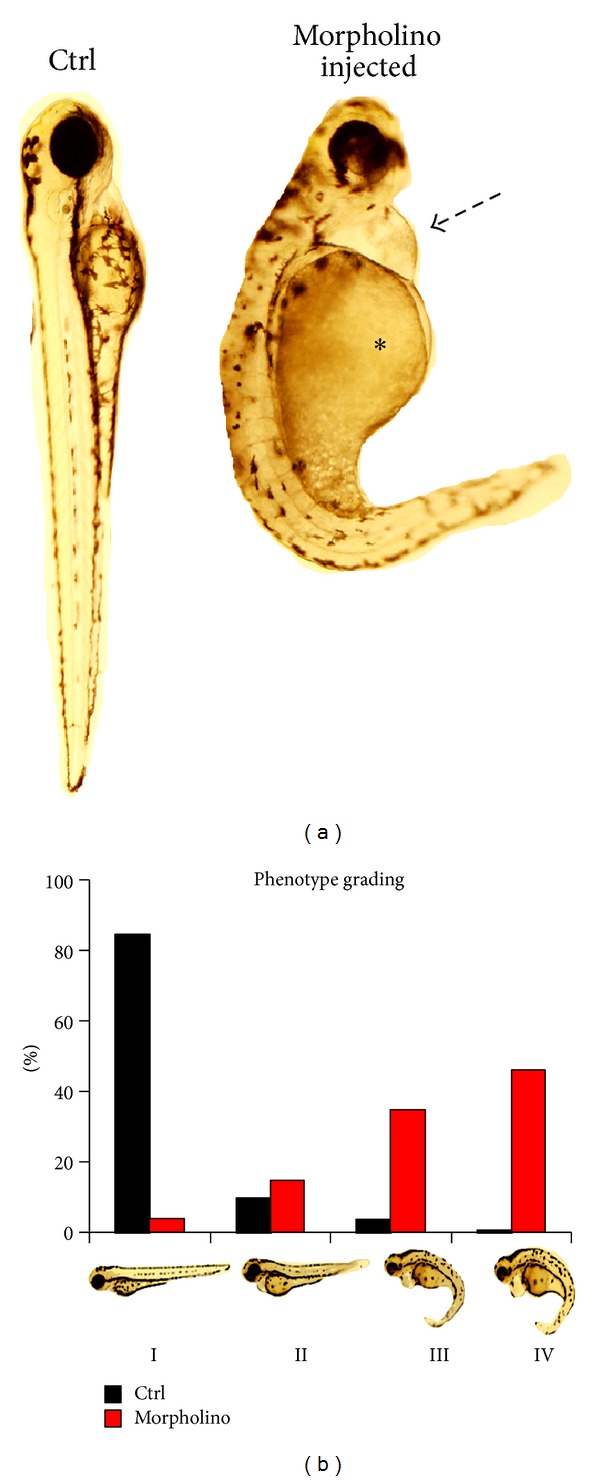
*Edema as a sign of kidney failure in zebrafish*. Edema in zebrafish is detected and rated as different grades of swelling in zebrafish embryos. (a) Modified fish (e.g., by morpholino knockdown) are examined at 120 hours postfertilization (hpf) and are compared to control (e.g., scrambled morpholino injected) fish. At that developmental stage a clear pericardial effusion (black arrow) and an edema of the yolk sac (asterisk) are visible. (b) Edema can be graded in four stages. Stage I: no signs of edema; stage II: mild edema; stage III: intermediate stage of edema; and stage IV: severe edema. Other features of the embryo such as curved or arched back deformities of the head structure, an absent or present swim bladder, and a sign of variable developmental delay, are highly variable, depend on the morpholino used, and need to be evaluated separately.

We classify zebrafish embryo edema phenotypes for qualitative and quantitative analysis in a range from phenotype I (PI) to phenotype IV (PIV) (Figure 1). To ensure that the quality of the eggs did not influence the phenotype development, it is always important to have a significant number of uninjected wildtype fish from the same clutch that were used for a specific injection. Uninjected and control morpholino-injected fish should develop a healthy looking slim shape (PI phenotype). In contrast, if the genetic modification leads to an edema as part of the phenotype, a higher percentage of knockdown morpholino-injected fish will develop severe generalized edema with pericardial effusion and yolk sac edema (PII to PIV).

This edema can range from mild edema (PII phenotype) to severe (PIII) or extremely severe (PIV). However, if a significant portion of genetically modified fish developed edema, the phenotype can only be interpreted as a first hint that the kidney is affected due to multiple causes of fluid accumulation in one or more cavities of the body. Generalized edema can be due to a rise in hydrostatic pressure caused by cardiac failure [41] a decrease in plasma oncotic pressure within blood vessels in nephrotic syndrome, or liver failure. Defining characteristics of human nephrotic syndrome are the following: significant proteinuria (>3.5 g/d/1.73 m² body surface area), hyperlipidemia, hypoproteinemia (≤2.5 g/dL) in the vascular system, and systemic edema. Renal sodium retention and changes in the variables of the starling equation are fundamental in the pathophysiology of the nephrotic syndrome [42]. Proteinuria is responsible for the development of hypoproteinemia and decreased plasma oncotic pressure. Therefore, plasma water translocates from the intravascular space into the interstitial tissues. Thus, it is important to confirm that the edema detected in zebrafish embryos is related to kidney disease. To differentiate between cardiac and renal phenotype, we established the following screening assays for zebrafish. 

## 6. Proteinuria Screening in Zebrafish

To establish that the observed edema phenotype is associated with the loss of high-molecular-weight proteins, we established the following assay systems: the tubular protein detection assay, the *FITC* labeled dextran assay (*FITC-eye-assay*), and the *Tg(l-fabp:DBP-eGFP) *assay (*Fabp-eye-assay*).

The zebrafish embryonic pronephros consists of two nephrons with glomeruli fused at the embryo midline and two pronephric tubules that connect the glomerulus to the pronephric ducts, that fuse just before the cloaca [43]. As in the mammalian kidney, the function of the pronephric tubules is to reabsorb essential proteins that are small enough to have passed through the glomerular filtration barrier. If the glomerular filtration barrier is compromised, normal or low-molecular-weight proteins as well as larger than 70 kD proteins can also pass through the glomerular filtration barrier and are then reabsorbed in the tubules. To document the reabsorption of high molecular weight proteins, the first assay we use is a tubular protein detection assay. We use the transgenic *wt1b:EGFP* zebrafish line that exhibits two orthologs found in teleost species such as human *Wilms tumor gene 1* (*WT1*), *wt1a*, and *wt1b* [44]. GFP expression in the *wt1b:eGFP* line can be observed in the pronephros starting at 17 hpf, at 35 hpf expression is detected in the pronephric glomeruli, tubules, and part of the ducts, and at 50 hpf the pronephros is fully functional and filtering blood. At this time the *wt1b* expression has migrated to the midline but has not fused, indicating that the expressing cells are found on the tubular pole of the glomerulus at the neck region of the proximal tubule [45]. GFP labeled *Wt1b* in the tubules of a 72-hour-old zebrafish is an excellent model to observe reabsorption of proteins in the tubules.

### 6.1. Tubular Protein Detection Assay

**Figure 2:**
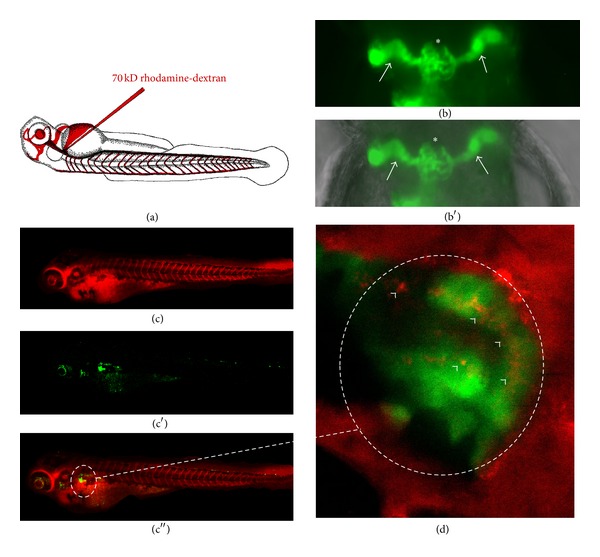
*Tubular detection of proteinuria*. The normal glomerular filter in a zebrafish embryo has a similar size selectivity compared to the glomerular filter in a mammalian kidney. Excessive amounts of high-molecular-weight proteins in the tubules would indicate a loss of this size selectivity and damage to the filter unit. To examine this we inject a 70 kD rhodamine-red-fluorescent labelled dextran (a) at 48 hours after fertilization into the cardinal vein of the control and morpholino-injected *wt1b*-transgenic fish. These fish express a green-fluorescent protein (b) in the glomerulus (asterisk in (b)) and in the proximal tubular part of the pronephros (white arrows in (b)) ((b) fluorescence view of a normal pronephros; (b′) fluorescent view merged with brightfield picture indicating the localization of the center of the pronephros in the pectoral fin region). The combination of both ((c)–(c′′), merged view enlarged in (d)) can be used to visualize reuptake of filtered high-molecular-weight dextran in the proximal tubular region (white arrowheads in (d)). Examinations can be performed in living, anaesthetized fish larvae allowing for serial examinations of the same animal over time.

To perform the tubular assay *Wt1b:EGFP*, zebrafish are mated, and the gene of interest morpholinos and the control solution are injected as described previously. The chorions are removed from the embryos manually with forceps at 48 hpf, and the embryos are ready for cardinal vein injection at 72 hpf. The anesthetized fish are positioned dorsally in a v-shaped agarose injectionmold. A 70 kDa rhodamine-labeled dextran is injected into the cardiac venous sinus. After injection, the embryos are moved into fresh embryo raising medium (ERM) and allowed to recover from anaesthesia. Embryos are placed in the 28.5°C incubator until imaging at 120 hpf. For imaging, the embryos are reanesthetized and imaging can be performed in live animals with a confocal microscope. If the glomerular filter has a barrier defect, significant amounts of red-fluorescent 70 kD rhodamine-dextran will be detectable inside the green fluorescent proximal tubular cells (Figure 2). This assay has several advantages: the fish are still alive and developing so imaging can be performed before or after the 120 hpf time point, tubules can be examined for glomerular leakage; normal development and the fusing zebrafish pronephros can be documented. Documenting the fusion of the pronephros is an important control, since severe developmental defects can be the sign of a developmental phenotype that affects normal kidney development.

### 6.2. Fluorescence Eye Assays

For differentiation between cardiac renal origin of the observed edema and we established eye assay models for indirect measuring of the integrity of the glomerular filtration barrier. Both systems share the concept that under normal conditions high-molecular-weight plasma proteins are retained in the circulation of the fish. If these plasma proteins are fluorescence labeled, they can be monitored over the retinal vessel plexus as representative locations for systemic fluorescence. A decreasing fluorescence level in the eye (e.g., after morpholino gene knockdown) supports our hypothesis of leakiness of the glomerular filtration barrier with loss of (fluorescence labeled) high-molecular-weight protein into the water. We use two different eye assay systems to detect if high-molecular-weight molecules pass through the glomerular filtration barrier: the *FITC*-eye-assay and *FABP*-eye-assay. 

**Figure 3:**
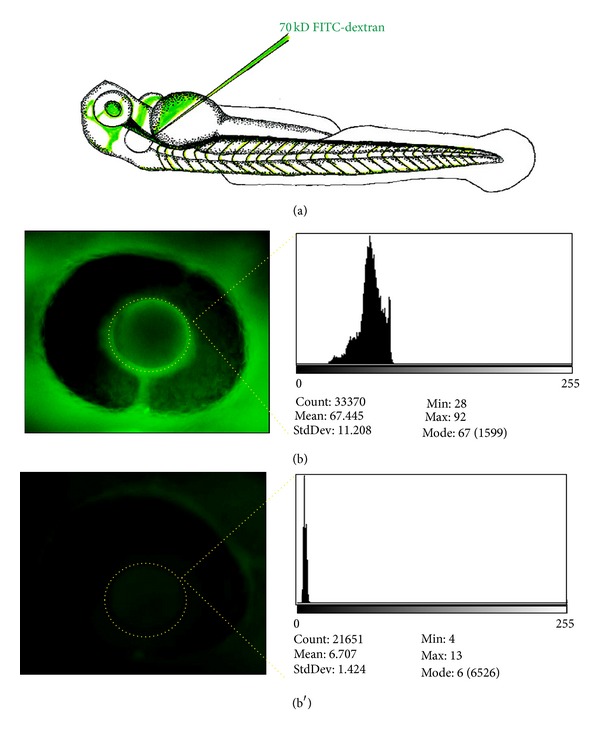
*Functional assay for glomerular filter integrity measuring systemic fluorescence over the retinal vessel plexus* (*FITC*-eye-assay). 70 kD *FITC*-labelled dextran is injected in the cardinal vein of anaesthetized morpholino and control-injected fish at 48 hours after fertilization (a). *FITC*-dextran level in the circulation is measured 24 hours after cardinal vein injection using ImageJ software as baseline value (b) and is additionally measured 48 hours after injection. If leakage of the 70 kD protein occurs at the filtration barrier, it is detectable by the significant loss of fluorescent dextran measured over time (b′), whereas in fish without leakage the protein is not lost and the measured fluorescence remains constant.

For the *FITC* assay, AB zebrafish can be mated, and the collected embryos are injected with both a targeted morpholino and a control morpholino at the 1-2 cell stage as described above. Dechorionated embryos at 48 hpf are then anesthetized and prepared for cardinal vein injection as described above. In this assay, we inject the fish with 4.6 nL of 70 kD *FITC* labeled dextran, allow them to recover and transfer them individually with 200 *μ*L of ERM into a 96 well plate, and maintain in a 28.5°C incubator. At 24 and 48 hours postinjection (hpi), images of the retinal vessel plexus of anesthetized fish are captured using an inverted microscope. The maximum fluorescence intensity is analyzed using the NIH ImageJ program in the fish retinal pupil [46]. We observe that fluorescence levels from 24 to 48 hpi uninjected wild type and control morpholino-injected fish remain steady or even increase. Increased intensity is likely to be due to continual uptake of the 70 kDa dextran [46]. However, if a genetically modified fish demonstrates a significant decrease in fluorescence intensity at 24–48 hpi, this indicates that the glomerular filtration barrier is compromised and is allowing the 70 kDa proteins to pass through (Figure 3). This technique can be used for morpholino injected as well as for any mutant fish line that has to be tested.

*Tg(l-fabp:DBP:EGFP)* fish, which were initially generated to visualize the blood brain barrier *in vivo* are used in the *Fabp*-eye-assay [47]. These fish express a vitamin D binding protein fused with the enhanced green fluorescent protein (*DBP-EGFP*) under the control of the liver-type fatty acid binding protein (*l-fabp*) promoter [47].

**Figure 4:**
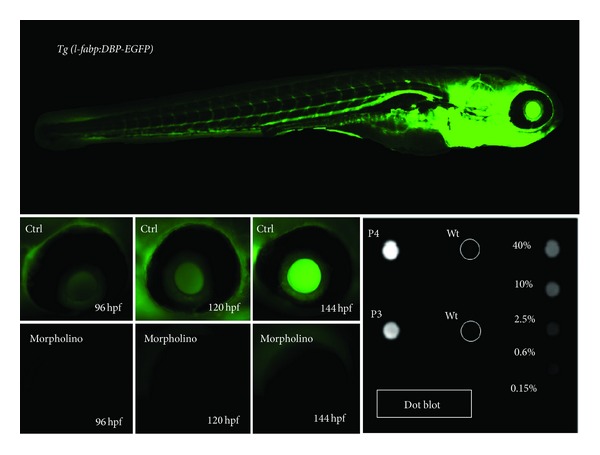
*Eye assay and dot blot for measuring glomerular filter integrity using transgenic l-fabp:DBP-eGFP* zebrafish. The l-fabp:DBP-eGFP transgenic zebrafish produces a green-fluorescent plasma protein. The transgene expression is driven by the *fabp*-liver promoter and leads to expression of a vitamin D binding protein fused with eGFP. The promoter becomes active at 2 days postfertilization that leads to the production and accumulation of fluorescent plasma protein that can be monitored over the retinal vessel plexus. If a morpholino injection leads to a compromise of the glomerular filtration barrier, fluorescence accumulation measured over the retinal plexus does not occur, and the eGFP that is lost via the kidney can be detected in the fish water, for example, using a dot blot approach.

The *DBP-eGFP* fusion protein has a molecular weight of approximately 78 kDa. Serial images of the retinal vessel plexus show a steady increase of fluorescence levels at 96 hpf, 120 hpf, and 144 hpf in a nonmorpholino-injected *Fabp Tg* fish. Similar to the assays above, the fish are mated and the eggs are injected at the 1-2 cell stage with a targeted morpholino or control morpholino and allowed to grow. A second injection step is not necessary in this fish line, making them a convenient model system to study effects in knockdown or overexpression systems. Following the imaging protocol used in the *FITC* assay, images of the eye are taken at 96, 120, and 144 hpf and analyzed using Image J. In contrast to the increasing fluorescent levels seen in the un-injected wild types and control morpholino-injected fish, the experimental morpholino-injected fish show a lower overall fluorescence that barely increases over time (Figure 4). 

The *FITC* and the *Fabp*-eye-assay systems both have advantages and disadvantages when compared with each other. The advantage of the *Fabp*-eye-assay is that cardinal vein injection is not performed. This injection takes practice to be accurate, creates a danger to the fish such that it can be harmed or die in the process, and is even a great challenge in knockdown or overexpression studies that create severe edema. The advantages of the *FITC*-eye-assay are that it can be performed on any strain of fish where the *FABP*-eye-assay is dependent on the *Fabp* transgenic fish and that the genetically mutant fish can be screened [48]. 

This technique has one important pitfall that has to be controlled. In the context pronephric development and embryonic blood flow, studies by Serluca et al. show that zebrafish mutants lacking blood flow as a result of cardiac defects fail to form a glomerular capillary tuft, indicating that while the phenotype is renal the underlying cause of the phenotype may be cardiac disruption [49].

To ensure that the observed phenotype is not caused by a cardiac dysfunction, the presence or absence of blood flow should be monitored in the tail region of the fish at 48 hpf and monitored until the end of the experiment using either the *FITC* or the *FABP* assay. These observations are important to consider in the final data analysis. 

*Detection of Proteinuria in Fish Water Assay.* The aforementioned assays only provide indirect evidence of proteinuria. The standard method of direct measurement of protein in urine from zebrafish is not feasible, because they live in an aqueous environment, so we developed an assay to measure proteinuria using a dot blot to concentrate proteins from the fish water, followed by immunoblotting that allows for sensitive, cost effective analysis of proteinuria. For this assay, we use the *l-Fabp:DBP-eGFP* transgenic zebrafish line mentioned above [47]. A positive signal for GFP on the dot blot of the fish water indicates that the 78 kD *DBP-EGFP* plasma protein has been excreted.

Here, we describe the dot blot method for detection of proteinuria in zebrafish embryos. Embryos injected with experimental or control morpholino at the 1-2 cell stage are allowed to develop in ERM overnight at 28°C. One day after fertilization viable embryos are transferred to fresh media. Two days after fertilization, embryos are dechorionated as necessary and assessed for proper cardiac function by visualizing erythrocyte movement under the light microscope to ensure that proper kidney development was not prevented by lack of blood flow [50]. Larvae fish are sorted into those with blood flow and those exhibiting no flow and are further separated into groups with either discernible endemic phenotype PIII/PIV, milder phenotype PII, or wildtype-like PI and are returned to 28°C with fresh ERM. The *l-fabp* liver promoter is turned on at 36 hpf and by 96 hpf the expression of *DBP-eGFP* is clearly visible in the circulation of the fish and will begin to be excreted in their urine if the filtration barrier of the pronephros is compromised. In order to maximize our ability to detect proteinuria in the fish water, we house the fish individually in 96-well plates with 250 *μ*L ERM/1 mM Tris pH 7.5 from 72 hpf to 168 hpf. ERM is supplemented with 1 mM Tris pH 7.5 to maintain solubility of *DBP:eGFP* [51]. Positive and negative *DBP:eGFP* protein controls in the 96-well plate are recommended. At 168 hpf 225 uL fish water per well is transferred to a fresh 96-well plate. Great care is taken not to wound the larva in the process of removing the fish water to avoid “protein pollution.” The fish water can be stored at −20°C until processing. 

Phenotype and flow groups are noted at the time of plating and reevaluated at the time fish water is collected at the end of the experiment. If a fish is wounded, its edema bursts during transfer, or it dies during the experiment, proteins will be released into the fish water and skew the dot blot results. We find inadvertent contamination of fish water is best controlled by segregating fish one per well and monitoring individual fish health and handling errors during the experiment. This enables the removal of individual fish from an experiment when problems are noted. To evaluate proteinuria, we use a dot blot apparatus to pull fish water (and the urine) through a nitrocellulose membrane by gravity filtration or light vacuum and then probe the membrane with an anti-GFP antibody. Water from fish may be pooled or analyzed individually. The severity of proteinuria can be assessed by comparing the level of signal from each well to signal from a quantitative protein extract made by 168 hpf *l-Fabp:DBP-eGFP* larva (Figure 4).

Other groups have reported methods for the detection of proteinuria in fish water. Hyvärinen et al. first reported directly measuring proteinuria fluorescence (excitation wave length 490 nm, emission wave length 535 nm, and measuring time 1 s) of fish water collected after 5 h incubation from larva injected with 500 kDa *FITC*-dextran into their cardinal vein [52]. Fluorescence levels in the fish water of two experimental morphants of transmembrane prolyl 4-hydroxylase (P4H-TM) were reported to be about 7.5- and 15-fold higher than levels found in water from control morphants. No data is given regarding the anticipated signal for the concentration of *FITC* injected, and raw fluorescence values are not stated so it is difficult to evaluate these data. Details regarding concentration or volume of the *FITC*-dextran injection, efforts to control *FITC* leakage from bleeding after injection or the volume of incubation, or whether fish were pooled for incubation were also missing from the text. The group of *Tryggvasson* turned to concentration of the fish water by TCA precipitation followed by SDS-PAGE to demonstrate proteinuria [53]. In their method, 100 control, WT, and morpholino-injected 96 hpf fish are each placed in 5 mL E3 water, which is changed twice and followed by a 24 h incubation at 28°C. At 120 hpf, after ensuring all fish were alive, 4 of 5 mL E3 water was TCA precipitated. SDS-PAGE was run using 12% Bis-Tris gel that was stained with Page-Blue Protein Staining Solution. Two bands were seen on the gel for both *nephrin* and *glcci1* knockdown embryos but not for wt or control morpholino-injected embryos. The 150 kDa and 70 kDa bands were identified by mass spectrometry and reported as a *vitellogenin* and a probable breakdown product of *vitellogenin*, respectively. *Vitellogenin* is a precursor to yolk proteins; its cleavage products are the main nutritional source for the developing embryo. Since* vitellogenin* is so abundant in the yolk and the yolk sacs of endemic fish can be quite fragile, although the prospect of this methodology is enticing, having *vitellogenin* as a read out for proteinuria of developing embryos is problematic. More recently *Zhou and Hildebrandt* reported a GFP ELISA method for proteinuria detection in zebrafish that employs an independently generated *l-Fabp:DBP-eGFP* transgenic line [54]. In this paper, they demonstrated that the biophysical properties of *(V)DBP-eGFP* closely resemble albumin. The aim of their study was to generate a low-labor intensive high-throughput method for the detection of proteinuria. Toward this end, they produced a dual transgenic fish by crossing *l-Fabp:DBP-eGFP* and *pod:NTR-mCherry* that allows for inducible nitroreductase podocyte injury through the introduction of metronidazole (MTZ) and the measurement of proteinuria through *DBP-eGFP*. To induce and assess proteinuria, twenty 3 dpf embryos per well were placed into 12 well-plates with zero to 10 mM MTZ in 1 mL 0.1% DMSO-E3 medium for 24 h. 100 *μ*L medium was analyzed for proteinuria using a GFP ELISA kit resulting in around 1200 pg/mL GFP from groups induced with 5 to 10 mM MTZ. Lower concentrations of MTZ did not provide a linear track of GFP readings by ELISA. Although no induction showed GFP levels near zero, 1 mM MTZ induction resulted in GFP near 300 pg/mL while 2 mM MTZ induction yielded GFP concentrations with error bars ranging from zero to 300 pg/mL. Overall this method appears promising for drug discovery when using a well-defined system starting with healthy larvae but may need work to produce quantitative results. When using the method to assess more unknown systems—as to whether knocking down a gene induces proteinuria—adequate cardiac function for glomerular fusion will still need to be assessed and care will need to be taken not to induce false positives through wounding of potentially fragile fish that will allow for distinguishing between disease versus technical issues.

## 7. Proteinuria versus “General” Vascular Leakage

**Figure 5:**
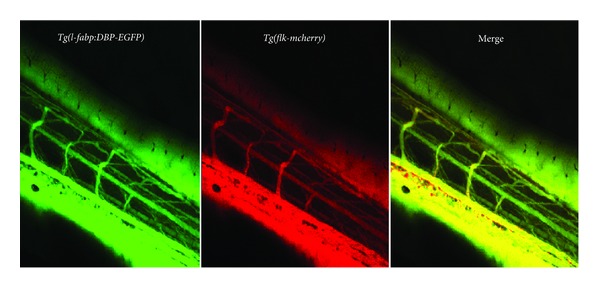
The *Tg(l-fabp:DBP-eGFP/flk-mcherry)* fish line is used to examine the integrity of the general vascular system of the fish. Normal development of the blood vessels can be examined as well as *DBP-eGFP*-fluorescence. If no vascular leakage is detected, the two fluorescent markers demonstrate a perfect overlap in the merged image.

Finally, it is important to ascertain if the observations made are specific for the glomerulus or if the genetic manipulation of the fish leads to general defects and leakiness of the vascular system. To accomplish this, we perform *in vivo* confocal imaging of a double transgenic *Tg(l-fabp:DBP-EGFP/flk-mcherry)* fish line, which has a red-fluorescent labelled vascular system driven by the promoter of the VEGF-receptor (*flk-mcherry*) in combination with *the l-fabp:DBP-EGFP*. This transgenic line has several advantages. First of all, normal development of the blood vessels can be examined, and we are able to visualize whether *DBP-EGFP* fluorescence leaves the vascular bed and diffuses into the interstitial space; if no vascular leakage is detected, the two fluorescent markers demonstrate a perfect overlap in the merged confocal images (Figure 5). If general vascular leakage is occurring, the overlap between *Fabp:DBP-eGFP* and *flk-mcherry* is reduced or absent. It is important to note these phenomena since changes observed in the eye assays could be attributed to a general effect on the vascular system. This does not exclude the presence of proteinuria but requires a more detailed structural workup of the vascular system in general and the glomerulus in particular.

## 8. Transmission Electron Microscopy

**Figure 6:**
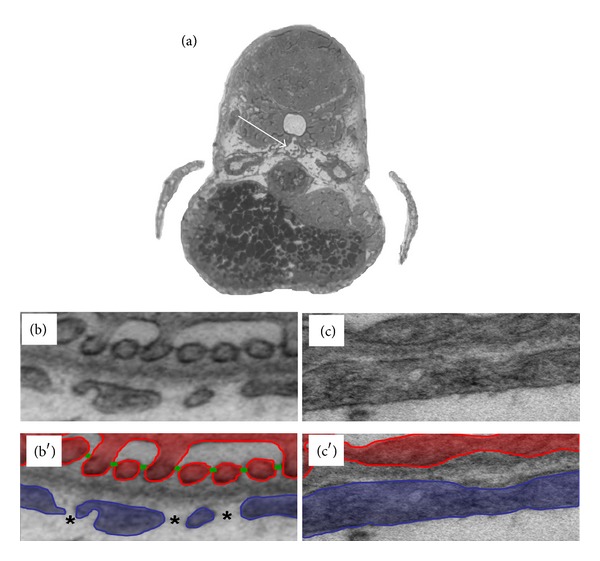
*TEM analysis to detect defects of the glomerular filtration barrier*. It is essential to perform a detailed structural analysis after proteinuria or leakage of the glomerular filtration barrier is detected, since any part of the filtration barrier (podocytes, GBM, or endothelial cells) could be affected. Experimental and control morpholino-injected embryos are embedded in epon blocks and trimmed to the glomerular region (white arrow in (a)). When the region is reached, ultrathin sections are prepared. Under normal conditions (b) the glomerular filtration barrier displays all features of a mammalian kidney with elaborate podocyte foot processes (red in (b′)) connected by slit diaphragms (green in (b′)) and a normal glomerular basement membrane and a fenestrated endothelium (blue in (b′), asterisk depicts fenestrae). Pathologic features after, for example, knockdown (c) include loss of elaborate foot process interdigitations and slit diaphragms (podocyte effacement) and/or loss of endothelial fenestrations (c′).

For further observation of the integrity of the renal barrier after eye assay and dot blot screening, we perform transmission electron microscopy (TEM) in collaboration with the Jackson Laboratory (Bar Harbor, ME, USA). Zebrafish embryos are fixed at 120 hours after fertilization and embedded in EPON (recipe/protocol from EMS, Hatfield, PA 19440, USA). We perform semi-thin (300 nm) and ultra-thin (90 nm) sectioning with a Leica UC-6 Microtome followed by transfer onto copper slit grids (EMS, Hatfield, PA 19440, USA) and imaging with a JOEL JEM-1230 transmission electron microscope (TEM). As we know from the early development of the zebrafish pronephros, the onset of the glomerular filtration occurs between 40 and 48 hpf [50]. We can generally identify the fused pronephric glomerulus with a diameter of about 40–80 *μ*m in the control fish. However, we frequently encounter difficulties identifying the pronephros when gene expression has been modified. In fish with stronger phenotypes, we observe that the renal structures are often relocated due to the severe generalized edema of the fish or have a nontypical morphologic structure. For detection of the glomerulus, we orient ourselves using familiar surrounding structures such as the notochord and the gut of the zebrafish embryo (Figure 6(a)). During TEM analysis, we take pictures from all detectable capillary loops at different magnifications (10.000x–30.000x). In most cases, it is obvious which part of the filtration barrier is affected but sometimes it requires a detailed grading of podocyte foot process integrity or swelling of the glomerular endothelium which requires high quality images of large capillary loop stretches in test animals and corresponding control (Figure 6(b)) from the same experiment.

## 9. Outlook

**Figure 7:**
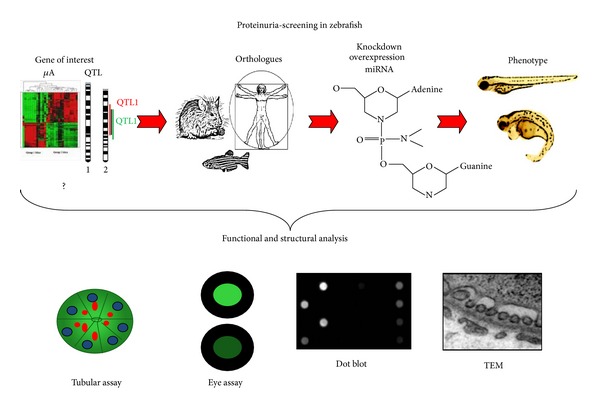
*Schematic overview of proteinuria screening in zebrafish embryos*. After identifying the zebrafish orthologue of the gene of interest, a morpholino knockdown is designed and injected in embryos. Phenotype analysis then offers the first indication of how the genetic manipulation affects the kidney. With functional assays and ultrastructural analysis compromise of the filtration barrier can be detected, and the affected structures can be identified.

We propose that, using the aforementioned techniques, it is possible to screen a large variety of genes in a short period of time and make solid statements regarding whether these genes are involved in the integrity of the glomerular filtration barrier (Figure 7). This screening system can be a helpful tool to discover novel genes, save time and resources and reduce the number of experimentally used rodents. Using these techniques, until now we were able to identify more than 25 novel genes formerly not described in glomerular biology.
